# Endophthalmitis and Mycotic Aneurysm: The Only Clues to Underlying Endocarditis

**DOI:** 10.5811/cpcem.2017.8.34723

**Published:** 2018-01-09

**Authors:** Guy Carmelli, Taylor Surles, Alisha Brown

**Affiliations:** * King’s County Hospital Center, Department of Emergency Medicine, Brooklyn, New York; † SUNY Downstate University Hospital of Brooklyn, Department of Emergency Medicine, Brooklyn, New York; ‡ University of Washington, Department of Emergency Medicine, Seattle, Washington

## Abstract

Infective endocarditis is a deadly disease that can present as a myriad of symptoms and thus its diagnosis can be missed. We present a case of infective endocarditis presenting as endogenous endophthalmitis and a ruptured mycotic aneurysm. This case illustrates both the complexity of infective endocarditis as a disease process and the more subtle diagnostic criteria as outlined by the Modified Duke Criteria.

## INTRODUCTION

Infective endocarditis (IE) remains a deadly disease, despite advances in modern medicine. The 30-day in-hospital mortality for IE is typically 15–20%^1^–^2^ and as high as 40% in the event that the patient is admitted to the intensive care unit.^3^ Proper, prompt diagnosis and management is imperative to minimize mortality. Furthermore, the wide range of presenting symptoms for this disease creates a diagnostic challenge. We present a unique case of a woman with IE who presented with both endogenous bacterial endophthalmitis and an intracerebral mycotic aneurysm that ruptured, causing a hemorrhagic stroke. Her presentation not only highlights the often diagnostically challenging nature of endocarditis, but also illustrates the underlying pathophysiology of this disease process.

## CASE REPORT

A 69-year-old female presented to the emergency department (ED) with a chief complaint of left-eye blurriness and discharge for two days. She described the discharge as yellowish, starting as a thin exudate that progressively became heavier over two days. She had associated malaise, fever, and multiple episodes of non-bilious, non-bloody emesis for one day. By the time she presented to the ED, she had lost vision in the affected eye. She denied contact lens use, eye pain with extraocular movements, or recent trauma to the eye.

Her past medical history was significant for hypertension and hyperlipidemia. She had no surgical history, no allergies, and no reported drug use. Her medications included amlodipine, hydrochlorothiazide, pantoprazole, and simvastatin, for which she reported compliance. On review of systems, she denied diarrhea, sick contacts, recent travel, fever, cough, sneezing, runny nose, headache/neck pain, chest pain/shortness of breath or abdominal pain.

**Image 1:**
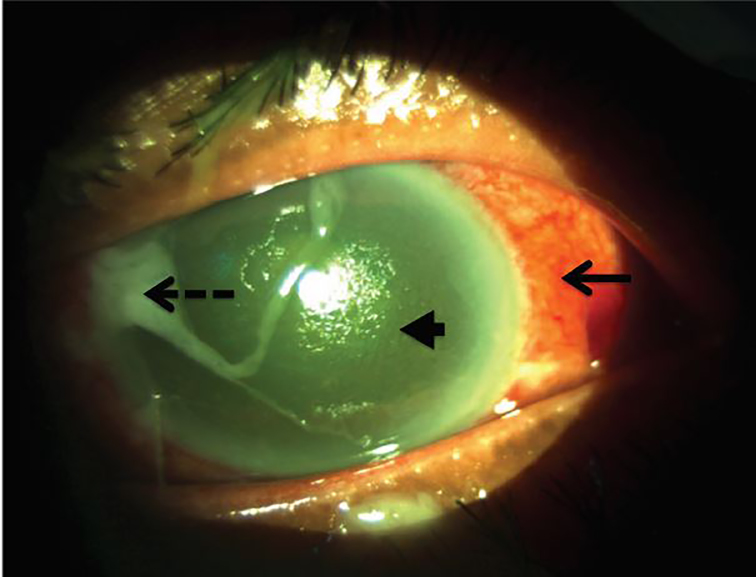
Image of patient’s left eye, taken by ophthalmology, demonstrating diffuse conjunctival injection without limbic sparing (solid arrow), mucopurulent drainage (dashed arrow) and a cloudy anterior chamber (arrow head).

Her triage vitals were as follows: blood pressure 125/71 mmHg, pulse 105 beats per minute, temperature 99.3 F (orally), respiratory rate 18 breaths per minute, and oxygen saturation of 95% on room air. Her initial ED exam showed an injected conjunctiva of the left eye with profuse mucopurulent drainage, normal pupillary response without an afferent pupillary defect, no photophobia, no proptosis and no pain with eye movements (Image 1). Her visual acuity was measured as no light perception. Her right eye had a normal exam. She had a normal cardiopulmonary exam, without any appreciable murmur. Her abdomen was soft and non-distended, but she had some minimal right upper quadrant pain. The rest of her exam, including an extremity, skin, and neurological exam, was within normal limits.

Labs were significant for a lactate of 1.7 mmol/L, a troponin I of 0.134 ng/ml (reference range normal <=0.10), and a white blood cell count of 3.09 K/uL with a predominance of immature neutrophils. Her electrolytes, renal function, liver function and lipase were normal. Her electrocardiogram (ECG) demonstrated sinus tachycardia with a few atrial ectopic beats, but no ischemic findings. An abdominal sonogram was performed by the radiologist to evaluate her right upper quadrant pain, and it showed no acute pathology. The patient was given an aspirin for her elevated troponin and Maalox for her abdominal pain. She was admitted to medicine for vomiting, fever without a source, possible non-ST-segment elevation myocardial infarction, and ophthalmologic evaluation.

While in the ED awaiting admission, ophthalmology evaluated the patient. On fundoscopic exam, the patient was found to have white fibrinous material with cell and flare in the anterior chamber, evidence of vitritis in the posterior segment and punctate intraretinal hemorrhages with central whitening thought to be Roth’s spots. Opthalmology’s findings were consistent with endophthalmitis. They obtained vitreous cultures and recommended a broad workup for an endogenous cause.

**Image 2:**
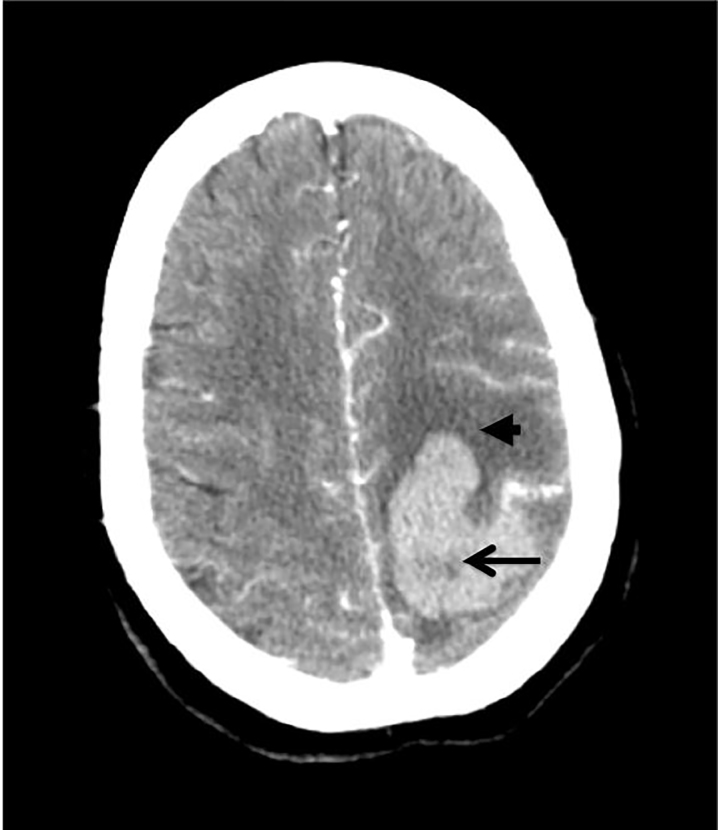
Computed tomographic angiography of the patient’s brain demonstrating left middle cerebral artery mycotic aneurysm with intracerebral hemorrhage (black arrow) and surrounding vasogenic edema (arrow head).

Blood cultures and serial troponins were obtained. While awaiting a transthoracic echocardiogram (TTE) in the ED, the patient developed new right upper extremity weakness and a stroke code was called. Computed tomography (CT) of the brain showed a left frontal lobe parenchymal hemorrhage with mild surrounding vasogenic edema and subarachnoid hemorrhage. CT angiography showed a mycotic aneurysm as the culprit lesion (Image 2), and the subsequent transesophageal echocardiogram (TEE) confirmed a valvular mass. The patient was treated for endocarditis with vancomycin and ceftriaxone intravenously. She was ultimately sent to physical rehabilitation for post-stroke care. The patient is now doing well and is awaiting valve replacement.

CPC-EM Capsule

What do we already know about this clinical entity?

Infective endocarditis (IE) is a deadly infection of the endocardial surface, most commonly the heart valves. Clinical manifestations can be extremely variable.

What makes this presentation of disease reportable?

Typical symptoms are pathognomonic for IE, such as Osler Nodules. However, our patient presented with endophthalmitis and intracranial hemorrhage, two relatively rare symptoms.

What is the major learning point?

Due to hematologic seeding, symptoms can arise in multiple organ systems at once. The Modified Duke Criteria provides a framework to stratify patients with possible endocarditis.

How might this improve emergency medicine practice?

When unusual symptoms present in multiple organ systems within a single patient, bacterial endocarditis must be on the differential.

## DISCUSSION

Our case report is unique in that this is a rare presentation of a classic disease. Our patient first presented with endophthalmitis and then had a hemorrhagic stroke from a mycotic aneurysm. Endogenous endophthalmitis is rare in the United States. One retrospective study by Okada et al. showed that in a large acute-care hospital over a 10-year period there were only 28 reported cases.^4^ Intracerebral mycotic aneurysms are almost as rare. In a retrospective review of 27 studies over 59 years, Ducruet et al. found only 287 cases.^5^ Therefore, the presence of both phenomena together in our patient is truly exceptional. It is important to first review both of these disease processes separately and then understand how they link to endocarditis.

### Endogenous endophthalmitis is an embolic event

Endophthalmitis is an infective process involving the anterior and posterior chambers of the eye and can be bacterial or fungal. It is further subdivided into exogenous or endogenous sources. Exogenous endophthalmitis is most common, typically resulting from a surgical procedure or direct ocular trauma.^6^ Endogenous endophthalmitis occurs in otherwise-healthy eyes, but infection is spread hematogenously from another infectious source in the body. Endogenous endophthalmitis accounts for 2–8% of all cases of endophthalmitis,^7^ with up to 40% resulting from IE.^4^

The diagnosis is largely clinical since the disease can rapidly progress and lead to permanent visual loss if treatment is not started before culture results are available. Key presenting symptoms are decreased vision (93%), conjunctival injection (81%), pain (75%) and lid swelling (33%). There typically is no fever or leukocytosis for endophthalmitis alone, and its presence should prompt an investigation for an endogenous source of infection.^6^ The hallmark of endophthalmitis is involvement in both the anterior chamber and posterior segments of the eye. Slit lamp examination may reveal cell and flare with or without a hypopyon in the anterior chamber. Vitreous inflammation and exudates may obscure the retina and hide the red reflex.^6^,^8^ Our patient came in with rapidly deteriorating vision secondary to infectious endophthalmitis. Given there was no history suggestive of an exogenous etiology, a broad search for an endogenous source was necessary.

### Mycotic Aneurysms are also embolic events

A mycotic aneurysm is an abnormal dilatation of an artery from bacterial involvement. This can be caused by inoculation of a previously weakened vessel wall, or by direct infection of a previously normal arterial wall from septic emboli, as is the case in IE. Studies show that up to 40% of patients with IE will have central nervous system involvement, with up to 3–10% of patients developing an intracerebral mycotic aneurysm (IMA).^9^,^10^ However, these numbers may be inaccurate because there are a number of silent IMAs that are only found on autopsy.^5^

The dilatation makes the arterial wall fragile and friable and therefore more prone to rupture. Clinical symptoms typically only present after the aneurysm has ruptured, causing a hemorrhagic stroke. Diagnosis is done primarily through neurovascular imaging and is typically only done after there is evidence of a stroke. Some may present first with fever (28%), headache (20%), hemiparesis (15%), or vomiting (9%).^5^ About 65% of people with IMA presented initially with IE. Therefore, most experts agree that anyone with IE who develops neurologic symptoms should undergo neuroimaging to rule out IMA.^5^

With regard to our patient, the next clue to underlying endocarditis came when she had an intracranial hemorrhage (ICH). While there is a broad differential diagnosis for ICH (including but not limited to hypertension, vascular malformation, or brain tumor), when presenting in the setting of simultaneous infectious endophthalmitis, septic emboli from an unknown source was at the top of the differential.

### Endocarditis is a syndrome- A review of the Modified Duke Criteria

IE is a challenging diagnosis to make because it can present as multiple, non-specific symptoms, and “textbook” presentations are rare.^11^ It was initially described by Sir William Osler in the late 1800s in his Gulstonian Lectures.^12^–^14^ It was not until 1994 that the Duke criteria were proposed as a means to diagnose IE. The criteria were later modified in 2000 (Table 1).^15^,^16^ These criteria are purposefully very broad because the pathophysiology of IE can produce symptoms in almost any organ system. In a study by Murdoch et al., the diagnosis of Roth’s spots, splinter hemorrhages, Janeway lesions, or Osler nodes occurred in 2%–8% of all patients.^17^ Rather, the most common presenting symptom was fever (96%) and either an elevated erythrocyte sedimentation rate (61%) or C-reactive protein level (62%),^17^ all of which are non-specific and could be related to a number of disease processes. However, one feature that differentiates IE from other systemic inflammatory responses is the presence of septic emboli. Vascular embolic events account for 17%–23% of complications from IE.^17^

At this point our patient met diagnostic criteria by Modified Duke Criteria for endocarditis. She had three minor criteria: a fever, an immunologic phenomenon (Roth’s spots on fundoscopy), and a vascular phenomenon (ICH). The TEE confirmed a valvular vegetation, fulfilling one major criteria, thus diagnosing endocarditis.

## CONCLUSION

We present a rare case of endocarditis that presented as rapidly progressing vision loss and hemorrhagic stroke from a mycotic aneurysm. The patient was aggressively treated with both intravenous and intravitreal antibiotics to reduce any further complications. She is alive and doing well to this day, awaiting definitive valvular replacement.

## Figures and Tables

**Table:** Modified Duke criteria. Remade from Li et al.^16^

Major criteria	Minor criteria
Positive blood culture with typical IE organism from 2 different blood cultures or persistently positive > 12 hours apart	Predisposing factor: known cardiac lesion or recreational intravenous drug use
Viridians- group *Streptococcus*	Microbiologic evidence: positive blood culture (not meeting major criterion) or serologic evidence of infection with organism consistent with IE but not satisfying major criterion
*Streptococcus bovis*	
HACEK group*	
*Staphylococcus aureus*	
Community-acquired Enterococci	
Evidence of endocardial involvement with positive echocardiogram defined as	Vascular phenomena: arterial emboli, pulmonary infarcts, intracranial hemorrhage, Janeway lesions, conjunctival hemorrhage, or mycotic aneurysm
Oscillating intracardiac mass on valve/supporting structures	
Abscess	
New valvular regurgitation	Fever of >/= to 38.0 C (100.4 F)
Dehiscence of prosthetic valve	
Single positive blood culture for *Coxiella burnetii* or anti-phase 1 IgG antibody titer >1:800	Immunological phenomena: Osler’s nodes, glomerulonephritis, rheumatoid factor, or Roth’s spots
Definite infective endocarditis (IE): 2 Major, 1 major/3 minor, or 5 minor	
Possible IE: 1 major/1 minor, or 3 minor criteria	

* HACEK group: Haemophilus species, Aggregatibacter species, Cardiobacterium species, Eikenella species, Kingella species.

*IE*, Infective Endocarditis; *C*, Celsius; *F*, Fahrenheit; *IgG*, Immunoglobulin G.

## References

[1] Cabell CH, Jollis JG, Peterson GE (2002). Changing patient characteristics and the effect on mortality in endocarditis. Arch Intern Med.

[2] Hoen B, Alla F, Selton-Suty C (2002). Changing profile of infective endocarditis: results of a 1-year survey in France. JAMA.

[3] Leroy O, Georges H, Devos P (2015). Infective endocarditis requiring ICU admission: epidemiology and prognosis. Ann Intensive Care.

[4] Okada AA, Johnson RP, Liles WC (1994). Endogenous bacterial endophthalmitis: report of a ten-year retrospective study. Ophthalmology.

[5] Ducruet A, Hickman Z, Zacharia B (2010). Intracranial infectious aneurysms: a comprehensive review. Neurosurg Rev.

[6] Yanoff M, Duker J (2014). Ophthalmology.

[7] Shrader SK, Band JD, Lauter CB (1990). The clinical spectrum of endophthalmitis: incidence, predisposing factors, and features influencing outcome. J Infect Dis.

[8] Sadiq MA, Hassan M, Agarwal A (2015). Endogenous endophthalmitis: diagnosis, management, and prognosis. J Ophthalmic Inflamm Infect.

[9] Sonneville R, Mourvillier B, Bouadma L (2011). Management of neurological complications of infective endocarditis in ICU patients. Ann Intensive Care.

[10] Silverman ME, Upshaw CB (2007). Extracardiac Manifestations of Infective endocarditis and their historical descriptions. Am J Cardiol.

[11] Baddour LM, Wilson WR, Bayer AS (2015). Infective endocarditis in adults: diagnosis, antimicrobial therapy, and management of complications. Circulation.

[12] Osler W (1885). The Gulstonian Lectures, on Malignant Endocarditis. Br Med J.

[13] Osler W (1885). The Gulstonian Lectures, on Malignant Endocarditis. Br Med J.

[14] Osler W (1885). The Gulstonian Lectures, on Malignant Endocarditis. Br Med J.

[15] Durack DT, Lukes AS, Bright DK, Duke Endocarditis Service (1994). New criteria for diagnosis of infective endocarditis: utilization of specific echocardiographic findings. The Am Journal Med.

[16] Li JS, Sexton DJ, Mick N (2000). Proposed modifications to the Duke criteria for the diagnosis of infective endocarditis. Clin Infect Dis.

[17] Murdoch DR, Corey GR, Hoen B (2009). Clinical presentation, etiology, and outcome of infective endocarditis in the 21st century. Arch Intern Med.

